# 

**Published:** 1886-11-20

**Authors:** 


PRICE ONE PENNY.

No. 8, Vol. I.
November 20th, 1886.

Per Annum, 6s. 6d., post free.

Monthly Parts, 6d.; post free, 7½d.

Ditto per Ann., 7s. 6d., post free.

### THE HOSPITAL

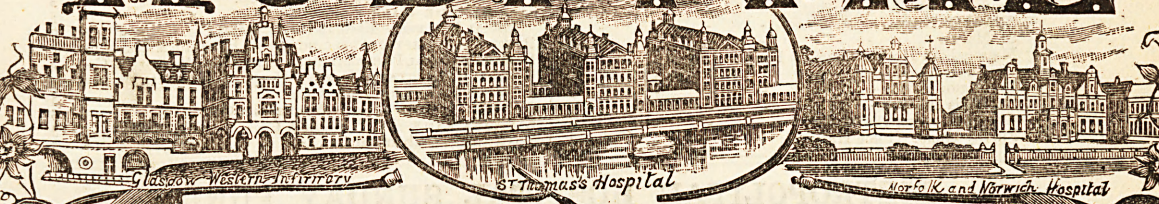

An Institution, Family, and Parochial Journal of

Hospitals, Asylums, & all Agencies for the Care of the Sick, Criticism & News.

